# Presenilin L166P Mutation, a Model of Familial Alzheimer's Disease, Leads to Early Onset Bone Loss

**DOI:** 10.1002/cph4.70097

**Published:** 2026-01-06

**Authors:** Vidyani Suryadevara, Connor J. Krehbial, Anuradha K. Valiya, Melinda Vang, Julian Balanta‐Melo, Pierre P. Eleniste, Sumana Posritong, Jung Min Hong, Katie Chester, Gabriel M. Pagnotti, Teresita Bellido, Monte S. Willis, Angela Bruzzaniti

**Affiliations:** ^1^ Department of Pathology and Laboratory Medicine, School of Medicine Indiana University Indianapolis Indiana USA; ^2^ Department of Biomedical Engineering Indiana University School of Medicine Indianapolis Indiana USA; ^3^ Department of Biomedical and Applied Sciences, School of Dentistry Indiana University Indianapolis Indiana USA; ^4^ Department of Endocrine, Neoplasia and Hormonal Disorders University of Texas‐ MD Anderson Cancer Center Houston Texas USA; ^5^ Indiana Center for Musculoskeletal Health, School of Medicine Indiana University Indianapolis Indiana USA; ^6^ Department of Anatomy, Cell Biology and Physiology, School of Medicine Indiana University Indianapolis Indiana USA

**Keywords:** aging, Alzheimer's disease, bone mass, mechanical function, presenilin 1, sex hormones

## Abstract

Accelerated bone loss has been reported in the early stages of Alzheimer's disease (AD) as indicated by reduced bone mineral density and increased fracture risk in these patients, compared to healthy individuals. In the present study, we investigated bone loss in mouse models of familial Alzheimer's disease harboring the Presenilin 1 (L166P) knock‐in mutation (PSEN1 KI), with or without the human amyloid precursor protein transgene (hAPP Tg+) known to induce brain amyloid pathology by 6 months. Female and not male 12‐month PSEN1/hAPP Tg+ mice exhibited reduced whole‐body bone mineral density and bone mineral content, compared to sex‐matched controls. Consistent with PSEN1 L166P driving the phenotype, female PSEN1 KI mice lacking the hAPP transgene also displayed low bone mass with a reduction in bone microarchitecture observed as early as 1 month of age. Correspondingly, PSEN1 KI mice exhibit reduced cortical and trabecular bone mass compared to age‐ and sex‐matched control mice. The loss of bone microarchitecture was largely attributed to a reduction in bone formation as indicated by decreases in serum P1NP levels and osteoblast ALP activity and mRNA expression in vitro. At the ages examined, PSEN1 KI mice exhibited increased follicle stimulating hormone (FSH) levels, which is known to cause a decrease in bone mass. Western blotting also identified both PSEN1 and amyloid‐beta protein expression in bone and brain tissue. Taken together, the data indicate that female‐specific bone loss in familial AD is potentially due to the direct actions of mutant PSEN1 in bone cells combined with systemic crosstalk caused by brain‐expressed PSEN1 L116P.

## Graphical abstract

Female‐specific bone loss in Alzheimer's disease mouse models: There is a significant alteration of bone phenotype during Alzheimer's disease. Using integrated imaging and biological assays, we observed changes in the bone microarchitecture, composition, and function in PSEN K1 and PSEN1/hAPP Tg+ mice, predominantly in the females.

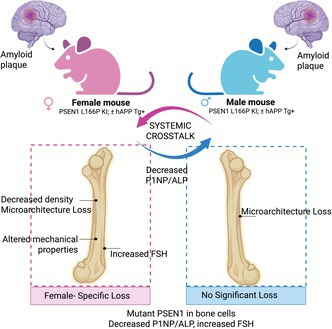

**Abbreviations**
AD: Alzheimer's disease
ALP: alkaline phosphatase activity
APP: amyloid precursor protein
Aβ: amyloid‐beta
BMC: bone mineral content
BMD: bone mineral density
Ct: cortical
CTX‐1: carboxy‐terminal collagen type‐1 crosslink fragments
DEXA: Dual‐energy X‐ray absorptiometry
E2: estrogen
FSH: follicle stimulating hormone
M‐CSF: Macrophage Colony Stimulating Factors
P1NP: procollagen type I N propeptide
PSEN1: presenilin 1
PTH: parathyroid hormone
Tb: trabecular
TRAP: tartrate‐resistant acid phosphatase
𝜇CT: microCT

## 1 Introduction

Alzheimer's disease (AD) is prevalent among 44 million people worldwide and is characterized by dementia and deterioration of cognitive capabilities. Emerging studies also demonstrate individuals with AD are more susceptible to falls and fractures and have a strong association with bone loss (Weller and Schatzker 2004). Longitudinal studies have identified that dementia patients have significant reductions in bone mineral density (BMD) (Tan et al. 2005). Furthermore, women with lower BMD have twice the risk of developing AD compared to women with higher BMD (Tan et al. 2005). These correlations are independent of other known risk factors for AD such as smoking, ApoE mutations, estrogen replacement therapy, age, and education level (Tan et al. 2005).

In studies investigating early‐stage dementia patients, AD patients demonstrate relatively lower mean BMD compared to the normal control group, correlated with the amount of brain atrophy and cognitive performance decline (Loskutova et al. 2009). A 2004 survey of the elderly in Canada showed that AD patients were more likely to have fallen during the past 12 months, to have sustained a hip fracture, and were more likely to have osteoporosis compared to individuals without AD. Notably, accumulating evidence suggests an increased risk of hip fracture in AD patients, independent of falling (Weller and Schatzker 2004) with some meta‐analyses reporting bone fractures increase up to two‐fold with AD (Melton 3rd et al. 1994). Further, fracture rates in women with dementia are nearly double those in the general population (32.7% versus 13.6%, respectively) (Johansson and Skoog 1996). Despite strong correlations between AD and bone loss, the underlying mechanisms remain unclear (Pinnamaneni et al. 2025).

AD is characterized by abnormal deposition of amyloid‐beta (Aβ) and neurofibrillary tangles of hyperphosphorylated tau in brain tissue (Rajmohan and Reddy 2017). Amyloid precursor protein (APP) is sequentially cleaved by β‐secretase and γ‐secretase to generate proteolytic fragments of Aβ (Weggen and Beher 2012). Aβ40 is the major cleavage product and the amyloid plaque deposition seen in brain tissue mainly occurs by the nucleate aggregation of Aβ42 fragments. Presenilin‐1 (*PSEN1*) serves as the core of the γ‐secretase complex which acts on APP to generate the Aβ fragments, Aβ42 and Aβ40. Human sequencing has established that familial AD can result from a single genetic mutation of *PSEN1* on chromosome 14, Presenilin‐2 (*PSEN2*) on chromosome 1 or amyloid precursor protein (*APP*) on chromosome 21. Familial AD accounts for 5% of all the AD and is inherited with a 50% probability. In several cases of familial AD, disease onset occurs as early as the third decade of life and is termed early‐onset AD (Singleton et al. 2000). Human *PSEN1* mutations are associated with an accelerated production of pathologic amyloid β peptide (Aβ42) and/or severely reduced Aβ40 levels, resulting in an increase in the Aβ42/Aβ40 ratio (Kelleher 3rd and Shen 2017).

Given the prominence of bone changes in AD patients, the current study investigated longitudinal changes in bone mass properties in the *Psen1* knock‐in mouse model of familial AD, PSEN1 L166P, to identify underlying mechanisms in AD‐associated bone changes.

## 2 Materials and Methods

### 2.1 Transgenic Mice

Two mouse models were used for these studies: PSEN1 KI/hAPP Tg+ mice and C57/BL6J (wildtype; WT) at 12 months of age and PSEN1 KI mice and littermate control mice lacking the knock‐in mutation (1–8 months of age). The PSEN1 KI/hAPP Tg+ mice have two copies of the genomic region encoding the PSEN1 L166P gene in endogenous tissues and contain the WT human APP transgene driven by an endogenous human promoter (from YAC) to mimic the distribution of these proteins in human disease (Vidal and Ghetti 2012; Lamb et al. 1993). When the PSEN1 KI mice were crossed with mice expressing the hAPP Tg+ (PSEN1 KI/hAPP Tg+), the resulting mice developed diffuse and compact Aβ deposits, cerebral amyloid angiopathy, and accumulation of intracellular Aβ with carboxyl‐terminal fragments of AβPP and Aβ42 peptide accumulation in the brain by 6 months of age (Vidal and Ghetti 2012). In contrast, PSEN1 KI mice lack the hAPP Tg+ but otherwise contain the same PSEN1 L166P knock‐in mutation. Previous studies of PSEN1 KI mice reported no amyloid pathology in brain tissue (Vidal and Ghetti 2012).

All mice were maintained in accordance with the Guide for the Care and Use of Laboratory Animals (National Institutes of Health, Bethesda, MD). PSEN1 KI/hAPP Tg+ and C57Bl/6 controls (wild type) were housed at the Indiana University School of Medicine (IUSM). PSEN1 KI mice and littermate controls were housed within the animal facility of Indiana University School of Dentistry (IUSD). All mice received regular chow diet (Harlan, Indianapolis, IN, USA) and water *ad libitum*, and were maintained on a 12 h light/dark cycle, according to USDA standards. Animals were euthanized according to IUSM/IUSD Institutional Animal Care and Use Committee (IACUC) approved procedures. The femur, tibia, and L4‐L6 vertebra of mice were dissected and cleaned of soft tissue. The left femur and L6 vertebra were then wrapped in saline‐soaked gauze and frozen at −20°C for μCT and/or 3‐point testing, while the right femur was fixed in 3.7% formaldehyde for 24 h and processed for histomorphometry.

### 2.2 Micro‐Computed Tomography (μCT) Imaging and Quantification of Bone

Ex vivo μCT scans of left femoral bones were acquired using a Skyscan 1172 (Bruker, Aarsellar, Belgium) scanner at 9 μm resolution using a 0.7‐degree angle increment, two frames averaged, through a 0.5 mm Al filter (V = 60 kV, I = 167 μA). Ex vivo μCT scans of L6 vertebrae were acquired at 6 mm resolution using the same instrument. The acquired μCT scans were reconstructed with NRecon (Version 1.4.7.2, 2005–11) and calibrated to hydroxyapatite‐mimicking phantoms (0.25 and 0.75 g/cm^3^ Ca‐HA). They were rotated to the optimal orientation using Dataviewer (Version 1.5.4.6, 2004‐11, 2012‐17), and the regions of interest were marked using CT Analyzer (CtAn) (1.18.4.0+, 2003–11,200). The cancellous bone was then segmented from cortical bone in the metaphysis region beginning 0.5 mm proximal to the growth plate and extending 1 mm proximally to obtain trabecular regions (~100 slices), which were analyzed using CTAn for the trabecular properties (Suryadevara et al. 2022). One mm of the femoral mid‐diaphysis (~100 slices) was analyzed for cortical properties using MATLAB (MathWorks, Natick, MA) (Powell et al. 2019). CtVol (Version 2.3.2.0, 2012‐16) was used to obtain the representative cortical and trabecular plots. The nomenclature reported follows the guidelines for assessing bone microarchitecture using μCT in rodents (Bouxsein et al. 2010).

### 2.3 Dual‐Energy X‐Ray Absorptiometry (DEXA)

Whole‐body DEXA scans were collected on isoflurane‐anesthetized mice using a PIXImus II densitometer (GE Lunar, Madison, WI). WT, PSEN1 KI mice were scanned at different months of age from 1 month to 8 months. From the whole‐body scans, bone mineral content (BMC) and bone mineral density (BMD) of femur and spine were calculated for the entire postcranial skeleton (minus the tail) using the GE Lunar ROI tools.

### 2.4 3‐Point Mechanical Testing

The left femurs, which were previously stored at −20°C in saline soaked gauze, were thawed to room temperature and hydrated in 0.9% saline. After μCT imaging, 3‐point bending was performed on the mid‐diaphysis region by loading them to failure at 2 mm/min and collecting the force versus displacement data at 10 Hz using a servo‐hydraulic test system (Test Resources Inc., Shakopee, MN, USA). Bones were aligned in an anterior–posterior direction with the upper contact area at the mid‐diaphysis region, while the lower contact points centered at the mid‐diaphysis region were separated by 7 mm. The cortical parameters of the bone cross‐sectional moment of inertia (I_AP_) and anterior–posterior diameter (c) measured using μCT were used to calculate the biomechanical properties of the femur using a custom MATLAB code previously developed by Dr. Joseph Wallace (Kohler et al. 2021). The yield was defined using the standard 0.2% strain offset method based on the stress–strain curve. I_AP_, c, the load, and deflection data were used to determine the structural and predicted tissue level properties by mapping the force: displacement and stress: strain, respectively, using the following beam‐bending equations.

$$\text{Stress}=\frac{F_{\mathrm{ac}}}{2I_{\mathrm{AP}}}=\sigma \left(\mathrm{MPa}\right)$$

$$\text{Microstrain}=\frac{6\mathrm{cd}}{a\left(3L-4a\right)}\times 10^{6}=\mu \varepsilon$$

In these equations, *F* is the force, *a* is the distance from the support to the inner loading point, *d* is the displacement, and *L* is the span between the outer supports. Force‐displacement and stress‐strain curve plots represent the material and structural properties determined from the three‐point bending test (Wallace et al. 2009).

### 2.5 Bone Histomorphometry

Histological staining with tartrate‐resistant acid phosphatase (TRAP)/toluidine blue (Sigma‐Aldrich, St. Louse, MO) and Von Kossa with McNeal counter stain were performed on the right femurs which were previously fixed in formalin and stored in 70% ethanol. Von Kossa/McNeal stained serial sections were analyzed for osteoblast number/bone surface and osteoid volume relative to mineralized bone surface (Pacheco‐Costa et al. 2014). TRAP and toluidine blue imaging were used to analyze osteoclast number and osteoclast surface per bone surface in the distal femur (Suryadevara et al. 2022). Histological analyses were performed using software from Bioquant Image Analysis Corporation (Nashville, TN). To maximize data consistency and minimize inter‐operator error, the same person completed each measurement at the same time using blinded specimens.

### 2.6 Osteoblast Alkaline Phosphatase Activity Assays

Osteoblasts were differentiated and assayed as per our previous publications (Huang et al. 2014; Posritong et al. 2018). Briefly, neonatal calvariae (1–3‐day pups) were freed of tissue and incubated in digestion buffer containing 0.1% collagenase A and 0.2% dispase II (ThermoFisher Scientific, Waltham, CA) dissolved in serum free αMEM for 15 min at 37°C. The cells were centrifuged at 2000 rpm. The digestions were repeated 3–5 times, each for 15 min. The supernatant from the first digestion was discarded, but all others were collected and pooled. Osteoblasts were cultured in αMEM containing 10% FBS for 7 days and passaged once before being used for experiments. Osteoblasts were seeded at 2 × 10^6^ cells per 1.9 cm^2^ wells and differentiated in media supplemented with 10 μM ascorbic acid and 50 μM β‐glycerol phosphate. After 7 days, the cells were washed with PBS and lysed in 1% Triton X‐100 supplemented with protease/kinase inhibitors, subjected to several freeze–thaw cycles, followed by centrifugation to collect the cell lysates. Alkaline phosphatase activity (ALP) was determined by the colorimetric conversion of p‐nitrophenol phosphate to nitrophenol (Sigma‐Aldrich, MO). Cell lysates were incubated with ALP substrate containing 2 mg/mL p‐nitrophenyl phosphate in 1.5 min alkaline buffer (Sigma‐Aldrich, MO), and the mixture was incubated for 50 min at 37°C in the dark, and the reaction stopped with 20 mM NaOH. Optical absorbance at 405 nm was read in a Thermax multiplate reader. ALP activity was normalized by total protein per well as determined using the BCA protein assay kit (ThermoFisher Scientific, Waltham, CA).

### 2.7 Osteoclast Differentiation

Osteoclast cultures were performed as previously described (Kang et al. 2019; Hong et al. 2024). Briefly, primary bone marrow‐derived macrophages (BMMs) from the tibiae and femora of 12‐week PSEN1 KI and WT mice were isolated by flushing with αMEM. After culturing for 24 h in αMEM containing 10% FBS, non‐adherent cells were collected and cultured in 6‐well plates at a density of 2 × 10^6^ cells/well in αMEM containing 10% FBS with Macrophage Colony Stimulating Factor (M‐CSF) (20 ng/mL) and Receptor Activator of Nuclear Factor Kappa‐B Ligand (RANKL) (80 ng/mL) to generate osteoclasts (PeproTech, NJ, USA). Fresh media was replenished every two days. The cells were fixed in 4% formaldehyde for 10 min and then stained for TRAP (Sigma‐Aldrich, St. Louse, MO). The number of TRAP‐positive cells with three or more nuclei and a red cytosol was counted. Representative images of TRAP‐positive osteoclasts were acquired using a Leica DMI4000 microscope.

### 2.8 Bone Turnover Markers and Hormone Assays

Whole blood from 4‐month and 8‐month PSEN1 KI mice was collected in serum separation tubes, allowed to clot for 20 min, and then centrifuged to separate serum, which was then collected and stored at −80°C. To minimize potential circadian rhythm effects, serum was collected at approximately the same time of day for each mouse. Serum parathyroid hormone (PTH), estradiol (E2), and follicle stimulating hormone (FSH) were assayed using commercial EIA/ELISA kits (mouse PTH EIA kit # EIAM‐PTH‐1, Raybiotech, Peachtree Corners, GA; mouse E2 estradiol ELISA Kit, ABIN1326835, Calbiotech, El Cajon, CA; FSH rodent ELISA kit, # KA2330, Abnova, Wuhan, China), according to manufacturer's instructions. Samples were run in duplicate using 50, 25, and 100 μL serum, respectively. Serum concentrations of procollagen type I N propeptide (PINP) and carboxy‐terminal collagen type‐1 crosslink fragments (CTX‐1) were determined as markers of in vivo bone formation and resorption, respectively, and were performed using enzyme immunoassay (EIA) kits, according to the manufacturer's instructions (AC‐33F1 and AC‐06F1, respectively, Immunodiagnostic Systems Ltd., Boldon, UK). Samples were run in duplicate using 5 and 25 μL serum, respectively.

### 2.9 Protein Extraction and Immunobloting

Tibiae were flushed and thoroughly cleaned of bone marrow and then snap frozen in liquid nitrogen. After pulverizing in liquid nitrogen, samples were suspended in modified RIPA lysis buffer (#89901 ThermoFisher, Waltham, MA) with protease and phosphatase inhibitors (#PC1010, Sigma, St. Louis, MO). The suspension was homogenized, pelleted, and the supernatant used as the bone protein lysate. Mouse brain tissue was similarly homogenized in modified RIPA lysis buffer with fresh protease and phosphatase inhibitors and homogenized in a Duvall glass homogenizer, incubated on ice for 10 min, centrifuged at 16,000 *g* for 15 min, and the supernatant collected. The protein lysate concentration was determined using the BCA protein assay kit (#23225, ThermoFisher Scientific, Waltham, CA). Twenty‐five μg/well protein was resolved on 4%–12% Bis‐Tris NuPAGE Protein gels (ThermoFisher Scientific, Waltham, CA), transferred to PVDF membranes, blocked with 1% bovine serum albumin and probed with primary antibodies (rabbit anti‐PSEN1, #529591 (1:1000), Calbiotech; mouse anti‐amyloid beta (Aβ)/APP monoclonal antibody clone 6E10, #9320–05 Signet Labs (1:500), mouse anti‐GAPDH (1:2000), Aldrich Sigma Co., St. Louis, MO) in TBS‐T overnight at 4°C. Membranes were washed in TBS‐T, then horseradish peroxidase‐conjugated secondary goat antibodies (Amersham, Arlington Heights, IL, USA) were added for 1 h at room temperature, followed by TBS‐T washes and visualization using PierceTM ECL Western Blotting substrate (#32209, ThermoFisher Scientific, Waltham, CA), according to the manufacturer's specifications. Membranes were then visualized on a ChemiDoc Imaging System (Bio‐Rad Inc., Hercules, CA).

### 2.10 Statistical Analysis

Statistical significance was determined using a two‐tailed Student's *t*‐test (*p* < 0.05) for two group comparisons and 2‐way ANOVA for interactions between genotype and sex. Data were analyzed using Microsoft Excel (Version 14.6.3, Redmond, WA) and Prism (Version 8.1.1 (330), GraphPad Inc., San Diego, CA). All results are presented as the mean data and standard deviation.

## 3 Results

### 3.1 Altered Bone Microarchitecture and Biomechanical Properties in PSEN1 KI/hAPP Tg+ Mice

**FIGURE 1:**
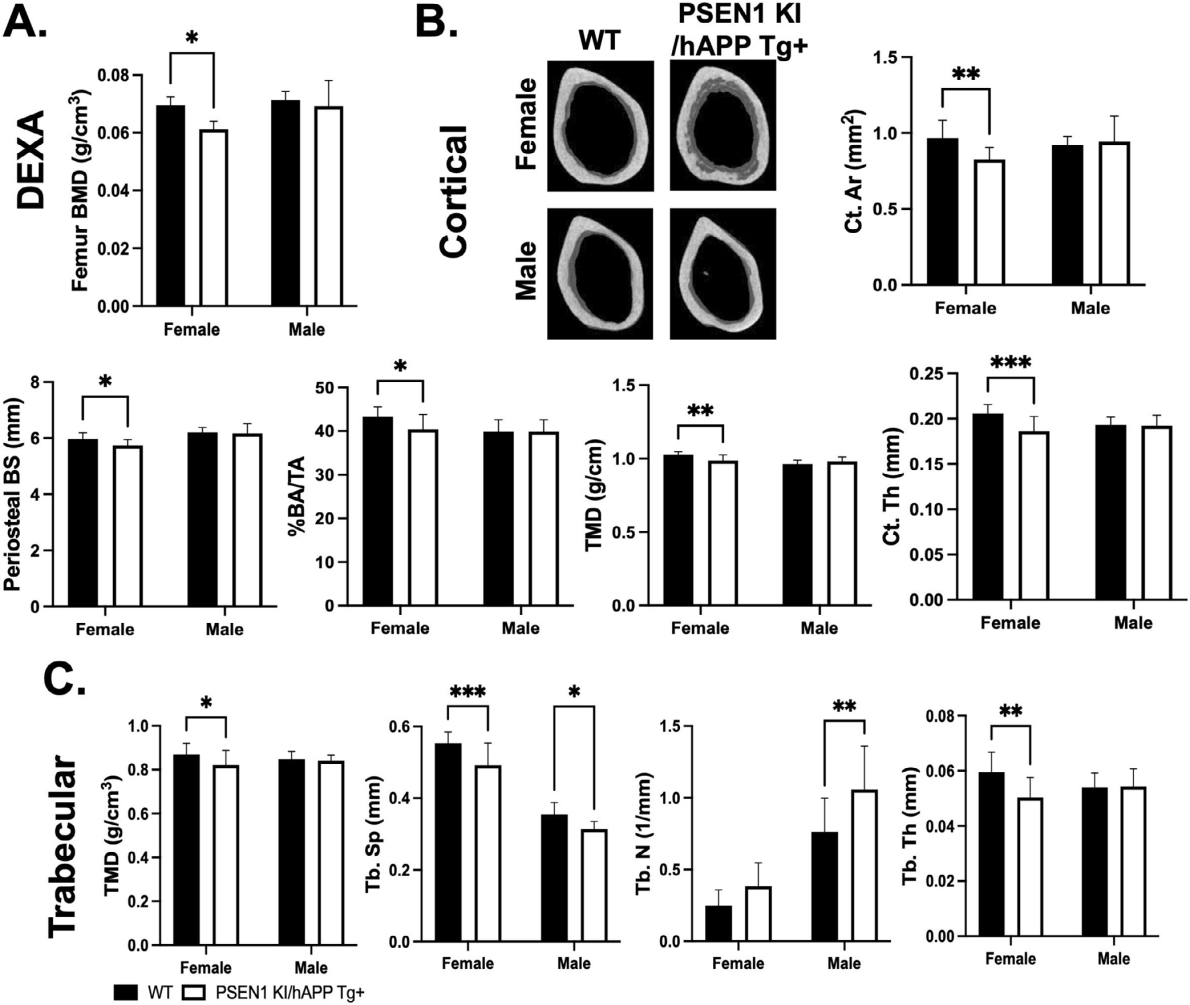
DEXA and μCT analysis of PSEN1 KI/hAPP Tg+ femur. 12‐month PSEN1 KI/hAPP Tg+ mice were analyzed by DEXA and μCT (Females: *N* = 12 WT, *N* = 12 PSEN1 KI/hAPP Tg+, Males: *N* = 10 WT, *N* = 9 PSEN1 KI/hAPP Tg+). (A) Bone mineral density (BMD) of femurs derived from male and female PSEN1 KI/hAPP Tg+ and WT mice. (B) Cortical properties analyzed by μCT from the mid‐diaphysis region of the femurs from male and female PSEN1 KI/hAPP Tg+ and WT mice and the representative cortical bone images. (C) Trabecular properties analyzed by μCT from the distal femur from male and female PSEN1 KI/hAPP Tg+ and WT mice. All data are displayed as mean and standard deviation. *p < 0.05, **p < 0.005.

Whole‐body DEXA analyses were conducted in 12‐month PSEN1 KI/hAPP Tg+ and control mice to determine skeletal properties. A significantly lower BMD was found only in female PSEN1 KI/hAPP Tg+ mice, compared with WT controls, whereas male PSEN1 KI/hAPP Tg+ mice did not display changes in DEXA data (Figure 1A).

Consistent with the DEXA results, 12‐month female PSEN1 KI/hAPP Tg+ femoral bones revealed significantly lower values for several cortical properties, including cortical area (Ct. Ar.), cortical thickness (Ct.Th.), periosteal bone surface, cortical bone per tissue area (BA/TA), and tissue mineral density (TMD), compared to WT femora as shown in Figure 1B; Table S1. However, no differences in BMD or any of the femoral cortical properties were observed between male PSEN1 KI/hAPP Tg+ and WT mice (Figure 1B, Table S1). Overall, changes in cortical bone measures were found in female, but not male PSEN1 KI/hAPP Tg+ femur at 12 months of age (Figure 1B). Analysis of cancellous (trabecular) bone in the distal femur of 12‐month female PSEN1 KI/hAPP Tg+ mice revealed a decrease in trabecular thickness (Tb.Th), trabecular separation (Tb.Sp), and tissue mineral density (TMD), compared to WT femora (Figure 1C, Table S1). In male 12‐month PSEN1 KI/hAPP Tg+ femora, lower Tb.Sp with a corresponding higher trabecular number (Tb.N) was observed (Figure 1C, Table S1).

When examining the vertebra of these mice, female PSEN1 KI/hAPP Tg+ were found to exhibit lower cancellous Tb.Th, with a corresponding higher Tb.N, compared to WT (Table S2). Similarly, male PSEN1 KI/hAPP Tg+ vertebrae had significantly lower trabecular bone volume fraction (BV/TV), trabecular number (Tb.N), and TMD at 12 months.

**FIGURE 2:**
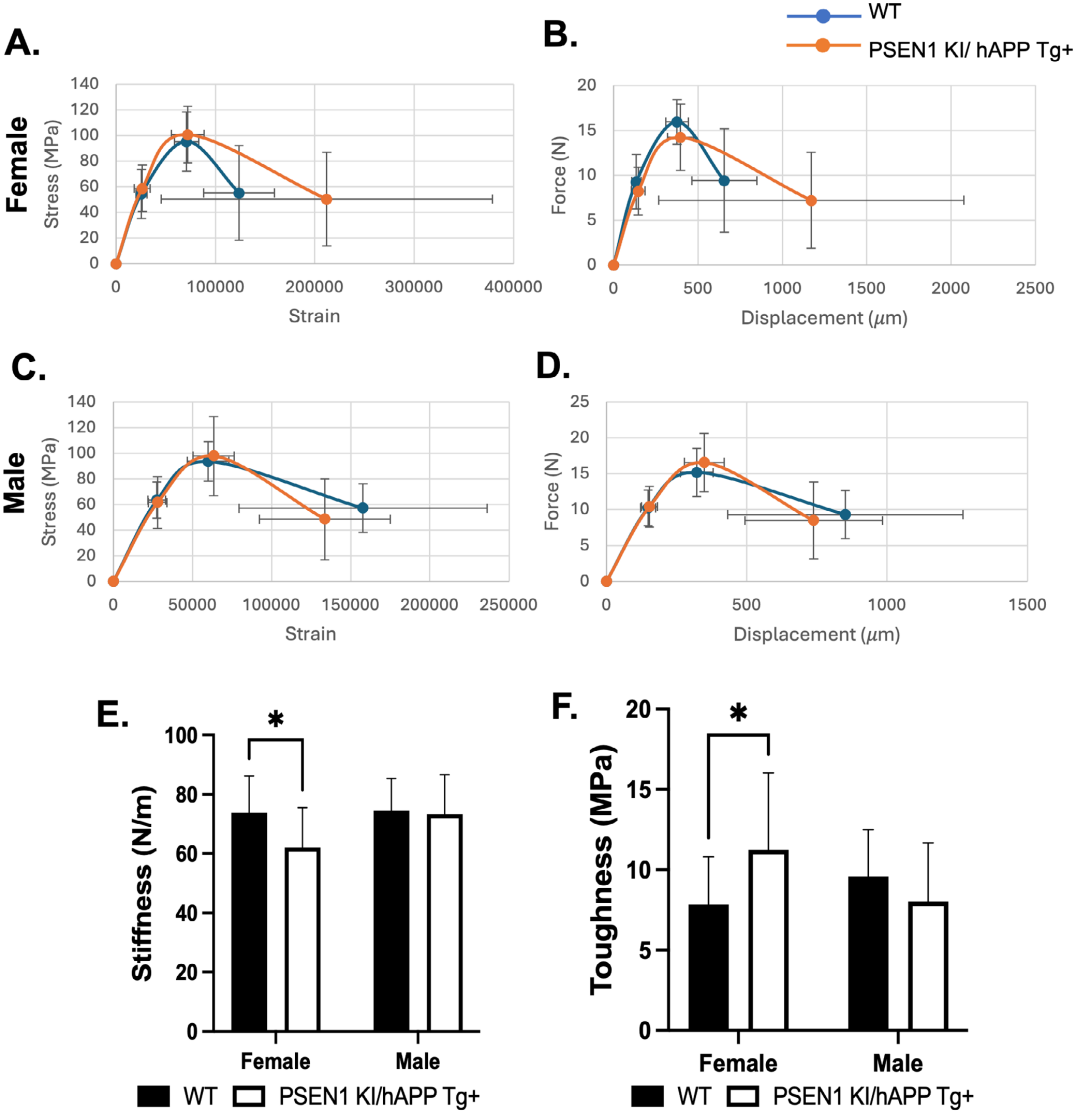
Biomechanical properties of female and male PSEN1 KI/APP Tg+ femur. Biomechanical properties were assessed using 3‐point bending tests of PSEN1 KI/hAPP Tg+ and WT femurs. (Females: *N* = 10 WT, *N* = 10 PSEN1 KI/hAPP Tg+, Males: *N* = 10 WT, *N* = 10 PSEN1 KI/hAPP Tg+). (A and C) Stress (MPa) versus strain curves for females versus males, respectively. (B and D) Force (N) versus displacement curves (μm). (E and F) Stiffness effects (N/mm) and Toughness (MPa) in female and male PSEN1 KI/hAPP Tg+ mice compared to controls. Data are displayed as mean ± standard deviations. Student's t‐tests were performed to determine significance between genotype and sex‐matched wildtype mice (C57BL/6J). *p < 0.05 compared to female WT.

Three‐point bending was performed to assess the mechanical properties of the femur. Studies revealed female PSEN1 KI/hAPP Tg+ femora exhibit lower stiffness and higher toughness compared to WT femora (Figure 2, Table S3). No changes were observed in any of the mechanical properties in the male PSEN1 KI/hAPP Tg+ femora, demonstrating female‐specific changes in the mechanical properties of 12‐month PSEN1 KI/hAPP Tg+ mice.

### 3.2 Early‐Onset Cortical Microarchitecture Deterioration in Female PSEN1 KI Mice

**FIGURE 3:**
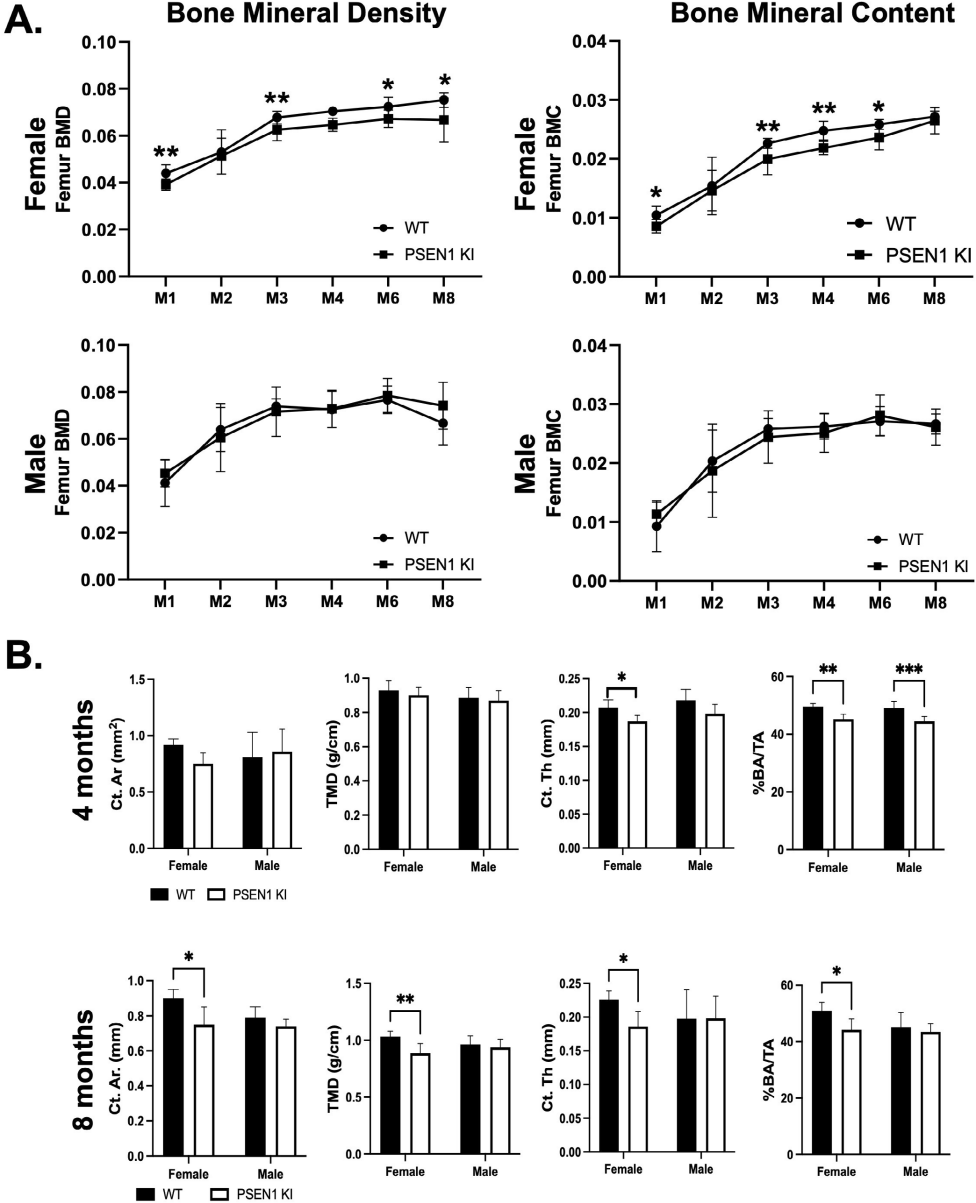
DEXA and μCT analysis of PSEN1 KI mice. (A) Serial longitudinal DEXA analyses were performed to measure BMC and BMD changes over several months (M) in PSEN1 KI and WT, male and female mice. (B) Cortical properties were analyzed by μCT in the femurs from 4‐month and 8‐month female PSEN1 KI mice. *N* = 5 mice per genotype/group. Data are mean and standard deviation. *p < 0.05, **p < 0.005.

Based on the significant alterations in the cortical and trabecular bone microarchitecture observed in 12‐month PSEN1 KI/hAPP Tg+ mice, we examined whether these bone changes were present in younger mice. However, the PSEN1 KI/hAPP Tg+ mouse line was discontinued, precluding analysis at younger ages. As an alternative, we used PSEN1 KI mice, which express the same L166P mutation but lack the human WT APP transgene. Using PSEN1 KI mice, we performed serial whole‐body DEXA analyses of female and male mice from 1 to 8 months of age (Figure 3A). Female PSEN1 KI mice showed significantly lower femoral BMC and BMD as early as one month of age, which was largely maintained through 8 months of age. In contrast, no changes in BMC or BMD at any of the ages examined were observed in male PSEN1 KI mice (Figure 3A).

Next we performed analysis by μCT of 4‐month female PSEN1 KI femora which revealed significantly lower Ct.Th and cortical bone area fraction (Ct.BAF), compared to WT mice (Figure 3B, Table S4). In addition, Ct.Th and Ct. Ar., Ct.BAF and TMD were lower in 8‐month female PSEN1 KI mice, compared to age‐matched WT mice (Figure 3B). The only significant change in male PSEN1 KI femora observed was in Ct.BAF, which was lower at 4 months, compared to male age‐matched controls (Figure 3B, Table S4). Overall, the cortical data for 4‐month and 8‐month PSEN1 KI mice were consistent with our 12‐month PSEN1 KI/hAPP Tg+ mice, suggesting a predominantly female‐specific effect on cortical microarchitecture in younger mice, with the cancellous bone phenotype displaying a later‐age onset.

### 3.3 Histomorphometric Assessment of PSEN1 KI Mice

Histomorphometry of 4‐month PSEN1 KI mice was performed to assess changes in osteoblast (Von Kossa) and osteoclast number (TRAP). Unexpectedly, the osteoblast and osteoclast parameters were largely unchanged in cancellous bone of PSEN1 KI and controls for both males and females. Of note, osteoid width was statistically lower in both female and male PSEN1 KI mice compared to sex‐matched littermates (Table S5).

### 3.4 Decreased Bone Formation Markers in Female PSEN1 KI Mice

**FIGURE 4:**
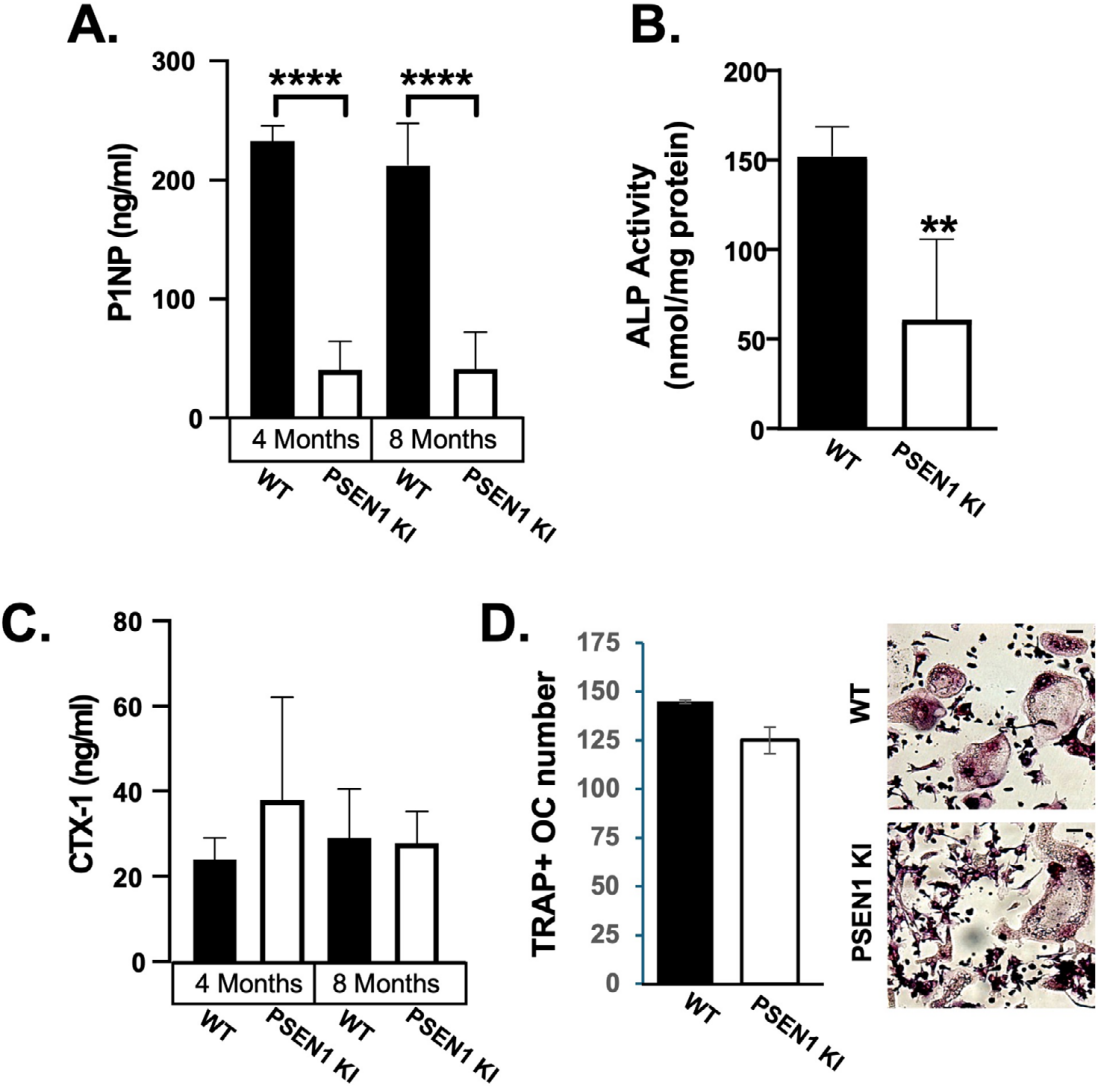
Effects of PSEN1 KI on osteoblast versus osteoclast activity in vivo and in vitro. (A) Bone formation activity assessed by ELISA of P1NP levels in serum in PSEN1 KI mice. (B) Osteoblast differentiation of neonatal calvariae derived cells from PSEN1 KI and WT mice cultured in vitro for 7 days and quantified for ALP activity. (C) CTX‐1 degradation products in serum were quantified by ELISA of PSEN1 KI mice at 4 months and 8 months. (D) Osteoclast differentiation of bone marrow cells from 6 to 8‐week‐old PSENI KI and WT mice. Cells from male and female mice were pooled. Mature, multinucleated osteoclasts were fixed, stained for TRAP activity and counted. Representative images of immature and mature osteoclasts. Scale bar indicates 20 μm. Data are mean and standard deviation. **p < 0.005.

Given that female 4–8‐month PSEN1 KI mice showed decreases in BMD and cortical bone properties, we sought to determine if the bone loss phenotype was due to underlying changes in bone formation and/or bone resorption activity. ELISA analysis of serum from our mice revealed significantly reduced bone procollagen type 1 N‐terminal propeptide (P1NP), a bone turnover marker, in 4‐month and 8‐month female PSEN1 KI mice, compared to control mice (Figure 4A). Consistent with decreased osteoblast activity, PSEN1 KI osteoblasts cultured in osteogenic media for 7 days exhibited reduced alkaline phosphatase (ALP) activity, an enzyme expressed in the osteoblast cell membrane that is critical for mineral deposition (Figure 4B). In contrast to the observed changes in bone formation markers, serum CTX‐1 levels, a marker of osteoclast resorption activity, did not reach significance in the male or female PSEN1 KI mice at the ages examined (Figure 4C). Similarly, no changes in osteoclast formation in vitro were observed (Figure 4D). Taken together, the observed decreases in osteoid width, P1NP levels and ALP activity, without changes in osteoclast activity, are consistent with an overall reduction in bone formation activity in PSEN1 KI mice.

### 3.5 FSH Levels Are Significantly Elevated in Female PSEN1 KI Mice

**FIGURE 5:**
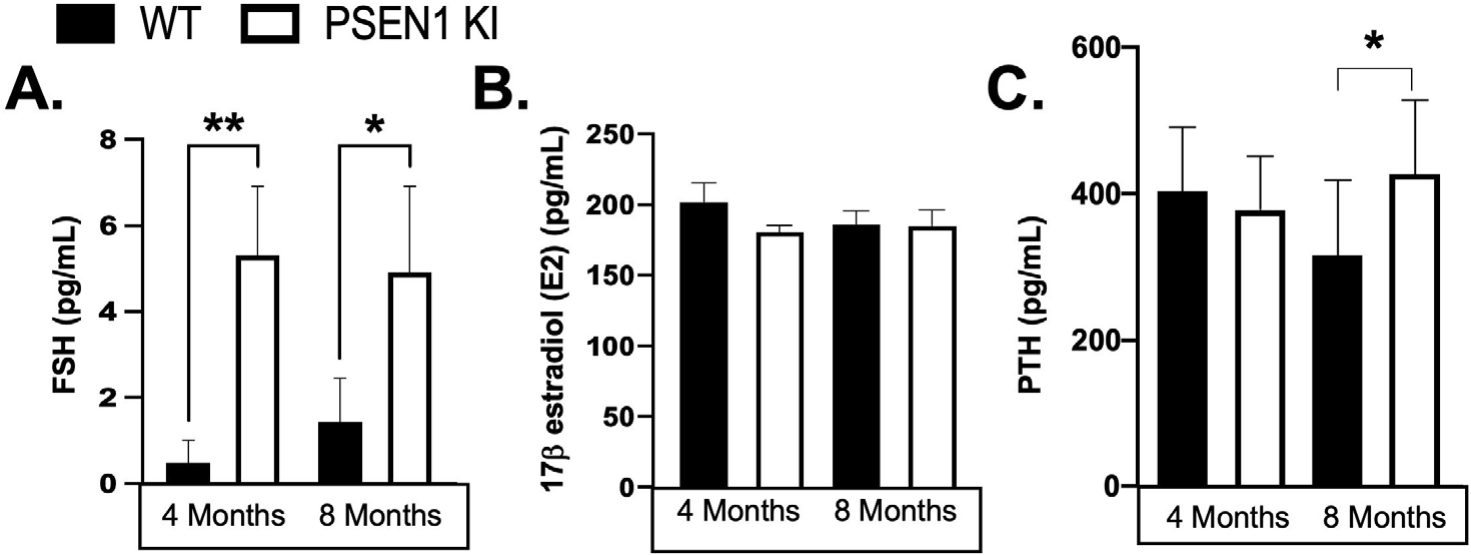
Serum hormone biomarker analyses of PSEN1 KI mice. Serum hormone levels of FSH, 17β‐estradiol (E2) and PTH were assessed in female 4‐month and 8‐month PSEN1 KI mice using specific EIA or ELISA kits. (A) FSH. (B) Estradiol (E2). (C) PTH. Data are displayed as mean and standard error of the mean. *p < 0.05, **p < 0.005 ****p < 0.0001.

To better understand the female‐specific bone loss phenotype of PSEN1 KI mice, we examined key circulating hormones known to affect bone mass. Specifically, elevated circulating follicle stimulating hormone (FSH) has been proposed as a mechanism of bone loss, complementing reduced 17β‐estradiol (E2) in menopause (Chin 2018). ELISA analysis of serum revealed significantly higher FSH levels in 4‐month and 8‐month PSEN1 KI mice, compared to their respective age‐matched controls (Figure 5A). In contrast, no statistical differences in E2 levels were evident in female PSEN1 KI mice, compared to age‐matched controls (Figure 5B). Elevated parathyroid hormone (PTH) is known to be associated with bone loss in hyperparathyroidism (Rolighed et al. 2014). However, although PTH levels were unchanged in 4‐month PSEN1 KI (Figure 5C), a small increase in PTH in 8‐month PSEN1 KI mice was observed, potentially reflecting an aging‐associated effect.

### 3.6 PSEN1 And Amyloid‐β Protein Are Expressed in Bone Tissue

**FIGURE 6:**
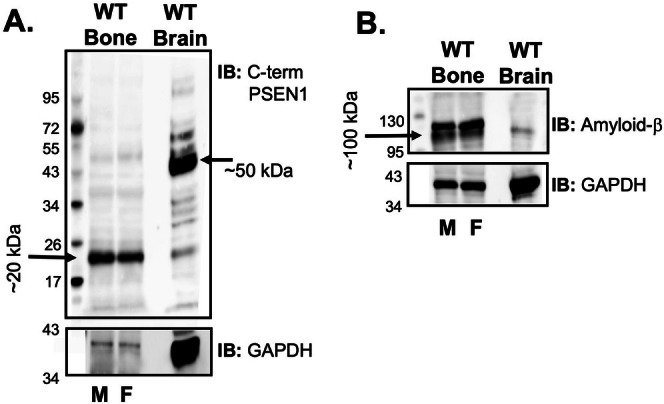
PSEN1 and amyloid‐β protein levels in bone versus brain tissue. (A) Expression of C‐terminal PSEN1 molecular weight species in bone tissue compared to brain tissue from male (M) and female (F) C57B/6 control (WT) mice. (B) Expression of amyloid‐β (APP) protein in bone and brain tissue from WT mice. Equivalent amounts of total protein were loaded for bone and brain tissues. GAPDH was used as the loading control. Molecular weight markers are indicated.

We performed Western blot analysis with an antibody to the C‐terminal domain of PSEN1 to compare baseline expression of PSEN1 as well as amyloid protein levels in bone versus brain tissue. Although PSEN1 was detected in both bone and brain tissues (Figure 6A), PSEN1 in bone displayed a different molecular weight profile than brain tissue. Specifically, PSEN1 immunoreactivity in bone was detected at ~50 kDa at low abundance, with the highest protein levels detected at ~20 kDa, contrasting with brain tissue that showed the highest immunoreactivity at ~45 kDa. The 50 kDa species represented full length PSEN1 whereas the ~20 kDa band likely represented a C‐terminal PSEN1 fragment, as previously described (Vidal et al. 2012; Moehlmann et al. 2002; Bentahir et al. 2006). Interestingly, Western blot analysis with an anti‐amyloid β antibody identified the ~100 kDa APP protein in WT bone lysates, which appeared to be more highly expressed in bone than in brain tissue (Figure 6B).

## 4 Discussion

In familial AD, PSEN1 L166P mutation elevates the production of the APP carboxyl‐terminal fragment (CTF), Aβ42, which is normally a minor Aβ species. A corresponding decrease in the Aβ40 fragment also occurs in a dose‐dependent manner in the brain of the PSEN1 KI/hAPP Tg+ mice (Vidal et al. 2012), consistent with in vitro studies of this mutation (Moehlmann et al. 2002). Increased amounts of the carboxyl‐terminal fragments of AβPP have been identified in cells expressing PSEN1 L166P (Moehlmann et al. 2002; Bentahir et al. 2006) and may result from reduced γ‐secretase activity rather than increased α‐ and β‐secretase processing of AβPP since both carboxy‐terminal fragments (Aβ42 and Aβ42) increase to the same extent in the cerebral cortex and hippocampus (De Jonghe et al. 2001; Qi et al. 2003). Consistent with the loss of the γ‐secretase activity, Aβ42/Aβ40 levels are elevated by 6 months of age in the brain of PSEN1 L166P KI/hAPP Tg+ mice (Vidal et al. 2012). Published literature has shown that APP promotes osteoblast survival and bone formation, specifically by altering mitochondrial ROS which contributes to apoptosis (Pan et al. 2018). Femurs from APP−/− mice were observed to have trabecular and cortical bone loss compared to their controls due to a reduction in bone formation. Further, it was observed that young adult Tg2576 mice with ubiquitous expression of APPswe demonstrate skeletal aging due to reduced bone formation and increased bone resorption (Xia et al. 2013). However, bone loss in young mice associated with PSEN1 mutations in familial AD has not previously been reported, and was the focus of the studies presented herein.

As shown in Figure 6, PSEN1 is expressed in bone tissue, displaying a different molecular weight profile compared to brain tissue. Unexpectedly, amyloid‐β protein was significantly more abundant in bone tissue compared to brain tissue. The higher amyloid‐related protein signal in bone likely reflects local APP/PSEN1 expression and differences in tissue composition and protein turnover rather than pathological amyloid accumulation. Taken together, the data implicate PSEN1 and amyloid‐β in the direct (cell autonomous) regulation of bone physiology as well as potential crosstalk between the bone and brain. Support for brain‐bone crosstalk comes from the TgAPPswe^OCN^ mouse model (Pan et al. 2021). These mice express APPswe in osteoblastic lineage cells (driven by osteocalcin) but have been shown to exhibit brain pathologies similar to human AD, including glial activation, elevated proinflammatory cytokines as well as altered learning and memory (Pan et al. 2021).

It is known that the PSEN1 KI/hAPP Tg+ mice develop brain amyloid pathology at approximately 6 months, including intracellular Aβ accumulation and increased AβPP C‐terminal fragments along with glial inflammation and neurite dystrophy (Vidal and Ghetti 2012). In contrast, PSEN1 KI mice lacking the APP are not known to develop amyloid pathology (Vidal et al. 2012). A limitation of the current study is that two different mouse models were used to characterize the bone phenotype in mice with the PSEN1 L166P mutation. However, both models used share the PSEN1 L166P mutation, allowing us to demonstrate that PSEN1 alone is sufficient to drive early bone loss, with the hAPP model showing persistence of the phenotype in the context of amyloid pathology in 12‐month mice. In the current study, we also observed reduced BMD in female PSEN1 KI mice as early as 1 month to 8 months of age (older mice were not examined). Furthermore, we observed reduced cortical bone properties in both 4‐month and 8‐month PSEN KI mice, compared to their age‐matched controls. Reduced bone mass was also observed in 12‐month female PSEN1 KI/hAPP Tg+ mice, with changes in both cortical and trabecular properties compared to the wild type. Pronounced loss of mineralization in the endosteal cortical zone observed by μCT suggests a region of hypomineralized cortical bone tissue which is mechanically weaker and more susceptible to local mechanical failure and reduced remodeling rate, compared to control mice. The endosteal cortical region normally has slightly lower mineralization than the periosteal cortex because of ongoing remodeling, but a pronounced loss indicates either increased resorption, impaired secondary mineralization, or both. Given the PSEN1 KI model did not display changes in osteoclast activity, it is likely that the low bone mineralization is due to decreased osteoblast activity. Further, a hypomineralized cortex will have reduced stiffness and strength for a given geometry, as indicated by the biomechanical assessments of the PSEN1 KI bones (Figure 2), even if overall cortical thickness or areal BMD were only modestly affected.

Interestingly, we observed increased trabecular number and reduced trabecular spacing in these mice, despite a reduction in the trabecular thickness, BMD, and cortical parameters. Given cortical bone mass is impacted by mechanical loading whereas trabecular bone largely responds to hormonal and metabolic cues, the results potentially reflect differences in the physiological roles of PSEN1 L166P in the different anatomical bone regions. The reduced bone stiffness in PSEN1 KI/hAPP Tg+ mice also indicates greater deformation of the bone under the same load (Morgan et al. 2018), whereas the higher toughness allows bone to dissipate more energy through microcracking and plastic deformation before complete fracture, which can reduce the likelihood of sudden, brittle breaks for any given mechanical insult. When stiffness decreases modestly while toughness increases, bone may better survive single high‐energy events (less brittle failure) but may be more vulnerable to deformity, fatigue damage, and fractures under repeated or chronic loading (Hernandez and van der Meulen 2017).

An emerging concept in age‐related osteoporosis is that elevated FSH, resulting from decreased estrogen, can directly regulate bone mass, further contributing to bone loss caused by estrogen deprivation (Sun et al. 2006; Wang et al. 2015). Although we did not detect suppression of circulating estrogen (E2) in our mouse model, significant increases in circulating FSH were observed, which remained elevated between 4‐month and 8‐month female PSEN1 KI mice (Figure 5A). These findings are consistent with a previous publication using 3–4 month female PSEN1 KI/hAPP Tg+ mice (Vidal et al. 2012).

The role of FSH in bone loss is far from clear. Elevated FSH has been detected in postmenopausal women, suggesting it negatively impacts BMD (Wang et al. 2015) and it was proposed that FSH contributes to postmenopausal osteoporosis by stimulating osteoclast differentiation (Wang et al. 2015) through the FSH receptor (FSHR) expressed on osteoclast precursors and mature osteoclasts (Chin 2018). However, other studies suggest elevated FSH increases bone mass (Wang et al. 2015; Ji et al. 2018), in part by increasing osteoblast surfaces (Wang et al. 2015). *FSHR* was reported to be expressed in both human and mouse brain, specifically within the cortex and various cells in the brain (Xiong et al. 2022). Systemic FSH was shown to trigger AD pathology and cognitive decline through C/EPBβ (Xiong et al. 2022), potentially explaining the higher incidence of AD in females. Our data support a combined mechanism involving both direct, cell‐autonomous effects of the PSEN1 L166P mutation in bone cells (early onset, reduced osteoblast activity in vitro, PSEN1 expression in bone) and systemic endocrine modulation, particularly elevated FSH, which likely explains the female specificity. This is consistent with the exaggeration of the AD pathology concurrent with higher FSH serum levels seen during menopause. At present, we cannot rule out the possibility that the elevated FSH in PSEN1 KI serum may have effects on other non‐bone tissues and organs that express the PSEN1 L166P mutation.

Investigation of PSEN1 expression (ENSG00000080815‐PSEN1) using the protein atlas revealed PSEN1 is expressed in the ovary and the parathyroid gland, which led us to investigate circulating E2 and PTH, respectively, as potential contributors to the observed low bone mass phenotype. As indicated above, we did not detect significant changes in E2 levels in 4‐month and 8‐month PSEN1 KI mice, ruling out E2 deficiency as a major mechanistic cause of the observed phenotype (Zaidi et al. 2018). Additionally, PTH was only minimally elevated in 8‐month PSEN1 KI mice and not changed in younger PSEN1 KI mice, ruling out hyperparathyroidism as a likely cause of low bone mass (Powell Jr et al. 2011; O'Brien et al. 2008). With respect to estrogen, although we did not observe changes in E2, it has been reported that female *PSEN1‐L166P* mice exhibit ovarian dysfunction (Vidal et al. 2012) making them infertile. The primordial follicles near the ovarian cortex of female *PSEN1‐L166P* (+/+) mice are largely composed of ovarian stromal elements that are not seen in their littermate controls (Vidal et al. 2012). Therefore, ovarian dysfunction cannot be ruled out in the mouse models investigated in our study.

Taken together, our findings reveal that PSEN1 L166P mutation leads to low bone mass that is seen as early as 1 month and persists in the ages examined in this study. Although the cellular and hormonal mechanisms require further investigation, our data suggest decreased bone formation contributing to the low bone mass phenotype, with the most significant findings being a decline in osteoblast activity in vitro and osteoid width and circulating PINP activity in vivo. Given histomorphometry showed only minimal changes in osteoclast or osteoblast parameters, we speculate that the major bone cell effects occurred during early growth and development. Furthermore, endocrine dysfunction, including elevated FSH, are likely contributors to the low bone mass phenotype caused by PSEN1 L166P mutation and familial AD.

## Funding

This work was supported by funds to Dr. Willis from the Physician Scientist Initiative (through the Lilly Endowment), the Strategic Research Initiative (IU School of Medicine) and the Bill & Melinda Gates Foundation, Seattle, WA. Dr. Bruzzaniti was supported by NIH grants R01AR060332 and R01AR080076 and by a professional development grant from the Indiana University School of Dentistry. Dr. Gabriel M. Pagnotti was supported by The Lawrence Family Bone Disease Program of Texas and the Cancer Prevention and Research Institute of Texas Grant 00011633 (Scholar of CPRIT Established Investigator Award RR190108: PI: T.A.G; Theresa A. Guise, MD is a CPRIT scholar in Cancer Research).

## Ethics Statement

All applicable international, national, and/or institutional guidelines for the care and use of animals were followed.

## Conflicts of Interest

The authors declare no conflicts of interest.

## Supporting information


**Table S1:** Cortical and Trabecular Femoral Bone Microarchitecture of Female and Male PSEN1 KI/hAPP Tg+ Mice. All data are displayed as mean data ± standard deviations. Student's *t*‐tests were performed to determine significance between sex‐matched experimental and wildtype mice (C57BL/6J). *N* = number of mice. Statistically significant values are indicated with asterisks and gray boxes *p < 0.05; **p < 0.005.

**Table S2:** Vertebral Trabecular Microarchitecture of Female and Male PSEN1 KI/APP Tg+ Mice. All data are displayed as mean data *±* standard deviations. Student's *t*‐tests were performed to determine significance between experimental and age‐matched wildtype mice (C57BL/6J). *p < 0.05; **p < 0.005. *N* = number of mice.

**Table S3:** Biomechanical Properties of Female and Male PSEN1 KI/APP Tg+ Femur. All data are displayed as mean data *±* standard deviations. Student's *t*‐tests were performed to determine significance between genotype and sex‐matched wildtype mice (C57BL/6J). *N* = number of individual mice. *p < 0.05; **p < 0.005 compared to wildtype.

**Table S4:** Trabecular and Cortical Bone Microarchitecture of 4‐Month and 8‐Month Female and Male PSEN1 KI mice. All data are displayed as mean data ± standard deviations. Student's *t*‐tests were performed to determine significance between experimental mice and age‐matched wildtype (WT) mice. *p < 0.05; **p < 0.005 (gray boxes). *N* = 5 mice/group. No significance was found in male cohorts.

**Table S5:** Static Histomorphometric Analysis of Female and Male PSEN1 KI Tibia. All data are displayed as mean data *±* standard deviations. Student's *t*‐tests were performed to determine significance between genotype and sex‐matched wildtype mice (C57BL/6J). Three serial sections were analyzed for each mouse. *N* = number of mice. *p < 0.05; **p < 0.005 compared to wildtype. All data are for 4‐month mice.

## Data Availability

The data that support the findings of this study are available from the corresponding author upon reasonable request.
