# Supplementary material for: Presenilin L166P Mutation, a Model of Familial Alzheimer's Disease, Leads to Early Onset Bone Loss
Source: Compr Physiol. 2026 Jan 6;16(1):e70097. doi: 10.1002/cph4.70097 (PMC12775720; doi:10.1002/cph4.70097)
Supplement: Supplementary file 1 — Table S1: Cortical and Trabecular Femoral Bone Microarchitecture of Female and Male PSEN1 KI/hAPP Tg+ Mice. All data are displayed as mean data ± standard deviations. Student's t‐tests were performed to determine significance between sex‐matched experimental and wildtype mice (C57BL/6J). N = number of mice. Statistically significant values are indicated with asterisks and gray boxes *p < 0.05; **p < 0.005. [file CPH4-16-e70097-s001.pdf]

Table S1. Cortical and Trabecular Femoral Bone Microarchitecture of Female and Male PSEN1 KI/hAPP Tg+ Mice. All data are displayed as mean data ± standard deviations. Student's t-tests were performed to determine significance between sex-matched experimental and wildtype mice (C57BL/6J). N=number of mice. Statistically significant values are indicated with asterisks and grey boxes *p<0.05; **p<0.005.

	**Female 12-month Wildtype** (N=12)	**Female 12-month PSEN1 KI/ APP Tg+** (N=12)	**Male 12-month Wildtype** (N=10)	**Male 12-month PSEN1 KI/ APP Tg+** (N=9)
Cortical bone microarchitecture				
Cortical bone area fraction (%)[$] (Ct.BAF)	43.26 ± 2.32	40.4 ± 3.404*	39.41 ± 1.91	39.95 ± 2.65
Total cortical surface area (mm^2) (Tt.Ar)	2.23 ± 0.2	2.05 ± 0.16	2.34 ± 0.17	2.36 ± 0.35
Marrow area (mm^2)	1.26 ± 0.1	1.22 ± 0.13	1.42 ± 0.14	1.41 ± 0.21
Cortical area (mm^2) (Ct.Ar)	0.97 ± 0.12	0.83 ± 0.08**	0.92 ± 0.06	0.94 ± 0.17
Cortical thickness (mm) (Ct.Th)	0.21 ± 0.01	0.19 ± 0.02**	0.19 ± 0.01	0.19 ± 0.01
Periosteal bone surface (mm) (Ps.pm)	5.97 ± 0.22	5.74 ± 0.21*	6.2 ± 0.18	6.17 ± 0.35
Endocortical bone surface (mm) (Ec.pm)	4.77 ± 0.18	4.7146 ± 0.19	5.08 ± 0.18	5.04 ± 0.31
Tissue mineral density (g/cm^3) (TMD)	1.03 ± 0.02	0.99 ± 0.04*	0.96 ± 0.03	0.98 ± 0.03
Trabecular bone microarchitecture				
Bone volume fraction (%) (BV/TV)	1.50 ± 0.78	3.56 ± 5.59	4.1 ± 1.22	5.84 ± 2.29
Trabecular thickness (mm) (Tb.Th)	0.06 ± 0.01	0.05 ± 0.01**	0.05 ± 0.01	0.05 ± 0.01
Trabecular separation (mm) (Tb.Sp)	0.55 ± 0.03	0.49 ± 0.06**	0.35 ± 0.03	0.31 ± 0.02*
Trabecular number (1/mm) (Tb.N)	0.25 ± 0.11	0.72 ± 1.16	0.76 ± 0.24	1.06 ± 0.301*
Trabecular pattern factor (1/mm) (Tb.Pf)	38.95 ± 7.36	37.37 ± 10.43	32.7 ± 5.45	31.63 ± 6.501
Tissue mineral density (g/cm^3) (TMD)	0.87 ± 0.05	0.82 ± 0.07*	0.85 ± 0.03	0.84 ± 0.03
Bone mineral density (g/cm^3) (BMD)	0.06 ± 0.01	0.07 ± 0.06	0.09 ± 0.01	0.104 ± 0.02

*[$]Ct.BAF = cortical cross-sectional area/total cross-sectional area (Ct.Ar/Tt.Ar)*100*
