# Supplementary material for: Presenilin L166P Mutation, a Model of Familial Alzheimer's Disease, Leads to Early Onset Bone Loss
Source: Compr Physiol. 2026 Jan 6;16(1):e70097. doi: 10.1002/cph4.70097 (PMC12775720; doi:10.1002/cph4.70097)
Supplement: Supplementary file 2 — Table S2: Vertebral Trabecular Microarchitecture of Female and Male PSEN1 KI/APP Tg+ Mice. All data are displayed as mean data ± standard deviations. Student's t‐tests were performed to determine significance between experimental and age‐matched wildtype mice (C57BL/6J). *p < 0.05; **p < 0.005. N = number of mice. [file CPH4-16-e70097-s003.pdf]

**Table S2. Vertebral Trabecular Microarchitecture of Female and Male PSEN1 KI/APP Tg+ Mice.** All data are displayed as mean data ± standard deviations. Student's t-tests were performed to determine significance between experimental and age-matched wildtype mice (C57BL/6J). *p<0.05; **p<0.005. N=number of mice.

| | **Female 12-month Wildtype** (N=5) | **Female 12-month PSEN1 KI/ APP Tg+** (N=4) | **Male 12-month Wildtype** (N=3) | **Male 12-month PSEN1 KI/ APP Tg+** (N=3) |
|---|---|---|---|---|
| Bone volume fraction (%) (BV/TV) | 2.87 ± 0.21 | 3.71 ± 0.82 | 4.1 ± 0.54 | 2.88 ± 0.22* |
| Trabecular thickness (Tb.Th) | 0.08 ± 0.004 | 0.06 ± 0.003** | 0.07 ± 0.009 | 0.06 ± 0.003 |
| Trabecular separation (Tb.Sp) | 0.6 ± 0.08 | 0.53 ± 0.08 | 0.43 ± 0.11 | 0.57 ± 0.06 |
| Trabecular number (Tb.N) | 0.36 ± 0.02 | 0.57 ± 0.12* | 0.63 ± 0.01 | 0.45 ± 0.05** |
| Trabecular pattern factor (Tb.Pf) | 8.1 ± 1.98 | 5.37 ± 2.54 | 10.5 ± 0.53 | 10.31 ± 2.84 |
| Tissue mineral density (TMD) | 7.33 ± 0.52 | 9.48 ± 2.09 | 10.48 ± 1.37 | 7.36 ± 0.56* |
