# Supplementary material for: Presenilin L166P Mutation, a Model of Familial Alzheimer's Disease, Leads to Early Onset Bone Loss
Source: Compr Physiol. 2026 Jan 6;16(1):e70097. doi: 10.1002/cph4.70097 (PMC12775720; doi:10.1002/cph4.70097)
Supplement: Supplementary file 3 — Table S3: Biomechanical Properties of Female and Male PSEN1 KI/APP Tg+ Femur. All data are displayed as mean data ± standard deviations. Student's t‐tests were performed to determine significance between genotype and sex‐matched wildtype mice (C57BL/6J). N = number of individual mice. *p < 0.05; **p < 0.005 compared to wildtype. [file CPH4-16-e70097-s002.pdf]

**Table S3. Biomechanical Properties of Female and Male PSEN1 KI/APP Tg+ Femur.** All data are displayed as mean data ± standard deviations. Student's t-tests were performed to determine significance between genotype and sex-matched wildtype mice (C57BL/6J). N=10 mice/group. *p<0.05; **p<0.005 compared to wildtype.				
	**Female 12-month Wildtype**	**Female 12-month PSEN1 KI/ APP Tg+**	**Male 12-month Wildtype**	**Male 12-month PSEN1 KI/ APP Tg+**
Yield force (N)	9.29 ± 3.02	8.25 ± 2.66	10.22 ± 2.48	10.39 ± 2.85
Ultimate force (N)	15.95 ± 2.5	14.24 ± 3.7	15.17 ± 3.37	16.57 ± 4.08
Displacement to field (μm)	134.76 ± 28.34	145.8 ± 41.49	148.08 ± 26.44	152.55 ± 28.01
Post yield displacement (μm)	521.41 ± 194.19	1024.81 ± 898.33	703.1 ± 422.29	585.32 ± 230.13
Total displacement (μm)	656.17 ± 192.67	1170.62 ± 904.38	851.18 ± 419.36	737.88 ± 244.96
Stiffness (N/mm)	73.75 ± 12.46	62.12 ± 13.42*	74.54 ± 10.83	73.36 ± 13.23
Work to yield (mJ)	0.71 ± 0.32	0.68 ± 0.42	0.85 ± 0.36	0.87 ± 0.39
Post yield work (mJ)	6.35 ± 2.5	7.99 ± 3.42	7.47 ± 2.53	6.43 ± 2.55
Total work (mJ)	7.06 ± 2.54	8.67 ± 3.51	8.32 ± 2.64	7.3 ± 2.54
Yield stress (MPa)	54.43 ± 19.16	58.74 ± 18.13	63.49 ± 14.22	61.63 ± 20.18
Ultimate stress (MPa)	95.26 ± 23.11	100.84 ± 22.02	93.6 ± 15.46	97.8 ± 30.73
Strain to yield (με)	25528.1 ± 5642.6	26397.4 ± 7987.5	27511.1 ± 5530	27958.4 ± 5905
Total strain (με)	123708.7 ± 35531.8	211919.9 ± 166583.4	157791 ± 78399.5	133635.6 ± 41274.4
Modulus (GPa)	2.32 ± 0.57	2.45 ± 0.5	2.51 ± 0.3	2.45 ± 0.78
Resilience (MPa)	0.77 ± 0.37	0.88 ± 0.55	0.98 ± 0.42	0.93 ± 0.43
Toughness (MPa)	7.85 ± 2.97	11.23 ± 4.78*	9.58 ± 2.92	8.01 ± 3.65
Failure force (N)	9.41 ± 5.77	7.21 ± 5.34	9.3 ± 3.36	8.49 ± 5.36
Failure stress (MPa)	55.27 ± 36.72	50.39 ± 36.43	57.2 ± 18.88	48.58 ± 31.59
Ultimate displacement (μm)	375.56 ± 67.38	396.92 ± 76.42	321.65 ± 58.24	347.88 ± 70.82
Ultimate strain (με)	70919.2 ± 12385.8	72138.4 ± 16407.1	59948.2 ± 13139.4	63392.9 ± 13102
