# Supplementary material for: Presenilin L166P Mutation, a Model of Familial Alzheimer's Disease, Leads to Early Onset Bone Loss
Source: Compr Physiol. 2026 Jan 6;16(1):e70097. doi: 10.1002/cph4.70097 (PMC12775720; doi:10.1002/cph4.70097)
Supplement: Supplementary file 4 — Table S4: Trabecular and Cortical Bone Microarchitecture of 4‐Month and 8‐Month Female and Male PSEN1 KI mice. All data are displayed as mean data ± standard deviations. Student's t‐tests were performed to determine significance between experimental mice and age‐matched wildtype (WT) mice. *p < 0.05; **p < 0.005 (gray boxes). N = 5 mice/group. No significance was found in male cohorts. [file CPH4-16-e70097-s004.pdf]

**Table S4. Trabecular and Cortical Bone Microarchitecture of 4-Month and 8-Month Female and Male PSEN1 KI mice.** All data are displayed as mean data ± standard deviations. Student's t-tests were performed to determine significance between experimental mice and age-matched wildtype (WT) mice. *p<0.05; **p<0.005 (grey boxes). N=5 mice/group. No significance was found in male cohorts.

| | **Female 4-mo WT** | **Female 4-mo PSEN1 KI** | **Female 8-mo WT** | **Female 8-mo PSEN1 KI** | **Male 4-mo WT** | **Male 4-mo PSEN1 KI** | **Male 8-mo WT** | **Male 8-mo PSEN1 KI** |
|---|---|---|---|---|---|---|---|---|
| ***Cortical bone microarchitecture*** | | | | | | | | |
| Cortical Bone Area Fraction (%) (Ct.BAF) | 49.55 ± 1.06 | 45.27 ± 1.69** | 50.99 ± 2.96 | 44.17 ± 3.85* | 49.2 ± 2.23 | 44.53 ± 1.65 | 45.19 ± 5.17 | 43.44 ± 2.87 |
| Total cortical surface area ($mm^2$) (Tt.Ar) | 1.61 ± 0.11 | 1.64 ± 0.08 | 1.77 ± 0.12 | 1.70 ± 0.09 | 1.81 ± 0.19 | 1.95 ± 0.24 | 1.77 ± 0.31 | 1.96 ± 0.35 |
| Marrow area ($mm^2$) | 0.82 ± 0.05 | 0.9 ± 0.05 | 0.87 ± 0.10 | 0.95 ± 0.05 | 0.92 ± 0.1 | 1.05 ± 0.1 | 0.96 ± 0.11 | 1.10 ± 0.15 |
| Cortical area ($mm^2$) (Ct.Ar) | 0.79 ± 0.06 | 0.74 ± 0.04 | 0.90 ± 0.05 | 0.75 ± 0.10* | 0.89 ± 0.1 | 0.91 ± 0.16 | 0.81 ± 0.22 | 0.86 ± 0.20 |
| Cortical thickness (mm) | 0.21 ± 0.01 | 0.19 ± 0.01* | 0.23 ± 0.01 | 0.19 ± 0.02** | 0.22 ± 0.02 | 0.21 ± 0.02 | 0.20 ± 0.04 | 0.20 ± 0.03 |
| Periosteal bone surface (mm) | 5.2 ± 0.16 | 5.22 ± 0.11 | 5.45 ± 0.22 | 5.36 ± 0.15 | 5.54 ± 0.23 | 5.67 ± 0.27 | 5.46 ± 0.48 | 5.69 ± 0.47 |
| Endocortical bone surface (mm) | 3.91 ± 0.12 | 4.05 ± 0.12 | 4.01 ± 0.24 | 4.17 ± 0.10 | 4.16 ± 0.22 | 4.39 ± 0.19 | 4.24 ± 0.3 | 4.54 ± 0.37 |
| Tissue mineral density ($g/cm^3$) (TMD) | 0.21 ± 0.03 | 0.2 ± 0.02 | 1.03 ± 0.05 | 0.89 ± 0.08* | 0.28 ± 0.06 | 1.14 ± 2.44 | 0.96 ± 0.07 | 0.94 ± 0.07 |
| ***Trabecular bone microarchitecture*** | | | | | | | | |
| Bone volume fraction (%) (BV/TV) | 4.46 ± 2.35 | 5.41 ± 1.71 | 3.59 ± 2.94 | 7.80 ± 3.26 | 14.72 ± 5.12 | 13.47 ± 5.26 | 8.10 ± 2.20 | 9.68 ± 3.34 |
| Trabecular thickness (mm) (Tb.Th) | 0.07 ± 0.009 | 0.06 ± 0.003 | 0.05 ± 0.008 | 0.05 ± 0.003 | 0.05 ± 0.007 | 0.05 ± 0.006 | 0.05 ± 0.008 | 0.05 ± 0.008 |
| Trabecular separation (mm) (Tb.Sp) | 0.29 ± 0.02 | 0.3 ± 0.02 | 0.36 ± 0.09 | 0.25 ± 0.07 | 0.2 ± 0.01 | 0.2 ± 0.02 | 0.24 ± 0.02 | 0.24 ± 0.03 |
| Trabecular number (1/mm) (Tb.N) | 0.98 ± 0.4 | 1.08 ± 0.29 | 0.77 ± 0.69 | 1.72 ± 0.68 | 2.76 ± 0.6 | 2.71 ± 0.69 | 1.66 ± 0.40 | 1.92 ± 0.55 |
| Trabecular pattern factor (1/mm) (Tb.Pf) | 42.05 ± 9.73 | 37.06 ± 6.28 | 39.5 ± 6.66 | 34.91 ± 4.92 | 22.18 ± 8.03 | 24.11 ± 7.52 | 31.68 ± 8.58 | 28.05 ± 9.24 |
| Tissue mineral density ($g/cm^3$) (TMD) | 0.74 ± 0.04 | 0.77 ± 0.02 | 0.80 ± 0.06 | 0.76 ± 0.03 | 0.78 ± 0.03 | 0.75 ± 0.03 | 0.81 ± 0.06 | 0.81 ± 0.07 |
| Bone mineral density ($g/cm^3$) (BMD) | 0.06 ± 0.02 | 0.06 ± 0.02 | 0.06 ± 0.03 | 0.09 ± 0.03 | 0.16 ± 0.05 | 0.15 ± 0.05 | 0.10 ± 0.03 | 0.12 ± 0.04 |
