# Supplementary material for: Presenilin L166P Mutation, a Model of Familial Alzheimer's Disease, Leads to Early Onset Bone Loss
Source: Compr Physiol. 2026 Jan 6;16(1):e70097. doi: 10.1002/cph4.70097 (PMC12775720; doi:10.1002/cph4.70097)
Supplement: Supplementary file 5 — Table S5: Static Histomorphometric Analysis of Female and Male PSEN1 KI Tibia. All data are displayed as mean data ± standard deviations. Student's t‐tests were performed to determine significance between genotype and sex‐matched wildtype mice (C57BL/6J). Three serial sections were analyzed for each mouse. N = number of mice. *p < 0.05; **p < 0.005 compared to wildtype. All data are for 4‐month mice. [file CPH4-16-e70097-s005.pdf]

Table S5. Static Histomorphometric Analysis of Female and Male PSEN1 KI Tibia. All data are displayed as mean data ± standard deviations. Student's t-tests were performed to determine significance between genotype and sex-matched wildtype mice (C57BL/6J). Three serial sections were analyzed for each mouse. N=number of mice. *p<0.05; **p<0.005 compared to wildtype. All data are for 4-month mice.

	**Female Wildtype** (N=6)	**Female PSEN1 KI** (N=6)	**Male Wildtype** (N=4)	**Male PSEN1 KI** (N=6)
TV	3.01 ± 1	3.48 ± 0.53	3.56 ± 0.47	3.99 ± 0.43
BV	0.16 ± 0.07	0.21 ± 0.08	0.49 ± 0.22	0.52 ± 0.22
BV/TV	0.06 ± 0.02	0.06 ± 0.02	0.14 ± 0.07	0.13 ± 0.06
BS	9.81 ± 4.19	11.13 ± 3.16	21.93 ± 5.27	25 ± 5.55
BS/BV	60.95 ± 8.39	57.14 ± 11.69	48.54 ± 11.8	51.83 ± 11.58
Tb.Dm (Rod)	0.07 ± 0.01	0.07 ± 0.02	0.09 ± 0.02	0.08 ± 0.02
Tb.N (Rod)	3.93 ± 0.36	3.76 ± 0.55	4.79 ± 0.21	5.01 ± 0.23
Tb.Sp (Rod)	0.19 ± 0.02	0.2 ± 0.04	0.13 ± 0.02	0.12 ± 0.02
OV	0.01 ± 0.01	0 ± 0	0.01 ± 0.01	0.01 ± 0.01
OV/BV	0.04 ± 0.03	0.01 ± 0.01	0.02 ± 0.02	0.17 ± 0.4
OS	1.67 ± 1.34	5.44 ± 7.31	1.88 ± 1.7	1.15 ± 0.84
OS/BS	0.15 ± 0.1	0.45 ± 0.57	0.1 ± 0.1	0.45 ± 0.96
O.Wi	3.78 ± 1.27	2.49 ± 0.37*	3.82 ± 0.8	2.18 ± 1.13*
N.Ob	157.67 ± 91.42	138 ± 35.68	204.75 ± 147.06	152.5 ± 72.91
N.Ob/BS	15.51 ± 5.36	13.16 ± 5.27	10.31 ± 7.9	6.34 ± 3.17
